# The genome sequence of the Swift Louse Fly *Crataerina pallida* (Latreille, 1812)

**DOI:** 10.12688/wellcomeopenres.20097.1

**Published:** 2023-10-09

**Authors:** Denise C. Wawman, George Candelin, University of Oxford and Wytham Woods Genome Acquisition Lab, Darwin Tree of Life Barcoding collective, Wellcome Sanger Institute Tree of Life programme, Wellcome Sanger Institute Scientific Operations: DNA Pipelines collective, Tree of Life Core Informatics collective, Darwin Tree of Life Consortium

**Affiliations:** 1 Edward Grey Institute, Department of Biology, University of Oxford, Oxford, England, UK; 2 Oxford University Museum of Natural History, Oxford, England, UK

**Keywords:** Crataerina pallida, Swift Louse Fly, genome sequence, chromosomal, Diptera

## Abstract

We present a genome assembly from an individual female *Crataerina pallida* (the Swift Louse Fly; Arthropoda; Insecta; Diptera; Hippoboscidae). The genome sequence is 177.0 megabases in span. Most of the assembly is scaffolded into 6 chromosomal pseudomolecules, including the X chromosome. The mitochondrial genome has also been assembled and is 21.57 kilobases in length.

## Species taxonomy

Eukaryota; Metazoa; Eumetazoa; Bilateria; Protostomia; Ecdysozoa; Panarthropoda; Arthropoda; Mandibulata; Pancrustacea; Hexapoda; Insecta; Dicondylia; Pterygota; Neoptera; Endopterygota; Diptera; Brachycera; Muscomorpha; Eremoneura; Cyclorrhapha; Schizophora; Calyptratae; Hippoboscoidea; Hippoboscidae; Ornithomyinae; *Crataerina*; *Crataerina pallida* (Latreille, 1812) (NCBI:txid452744).

## Background

The Swift Louse Fly *Crataerina pallida* is a haematophagous ectoparasite of birds. It is usually found on the swift *Apus apus*, Alpine swift *A. melba*, or pallid swift *A. pallidus*, and occasionally on Hirundines or Passerines ( 12, 1981; Hutson, 1984; Oldroyd, 1966). Its range is restricted to Europe with a few records from South Africa ( GBIF Secretariat, 2022) presumably from overwintering Swifts. The first record of the species in Britain or Ireland is from Selborne, Hampshire in 1774 ( White, 1789).

Swift Louse Flies are highly adapted to their lifestyle: specially adapted feet enable them to cling to their host ( Petersen *et al.*, 2018) and flattened bodies and reduced functionless wings help them hide amongst feathers ( Hutson, 1984; Oldroyd, 1966). Whereas most flies lay eggs, *C. pallida* is larviparous. A single larva is fed on a secretion from a gland in the female’s uterus and is only deposited into the hosts’ nest when it is ready to pupate ( Hutson, 1981; Oldroyd, 1966). Despite their large size compared to their hosts, parasite aggregation, and infestation rates of up to 70% of adult swifts and over 90% of nests, there is no evidence that they harm their hosts or influence reproductive success ( Hutson, 1981; Lack & Lack, 2018; Lee & Clayton, 1995; Walker & Rotherham, 2010).

This is the first complete genome of a member of the family Hippoboscidae to be published, although complete mitochondrial genomes are available for some species ( Li *et al.*, 2022; Liu *et al.*, 2017; Wang *et al.*, 2021).

We present a chromosomally complete genome sequence for *Crataerina pallida*, based on one specimen hatched from a puparium collected from a swift nest in the tower of Oxford University Museum of Natural History, where swifts have been monitored since 1947 ( Lack & Lack, 2018; Lack, 1951).

## Genome sequence report

**Figure 1.**
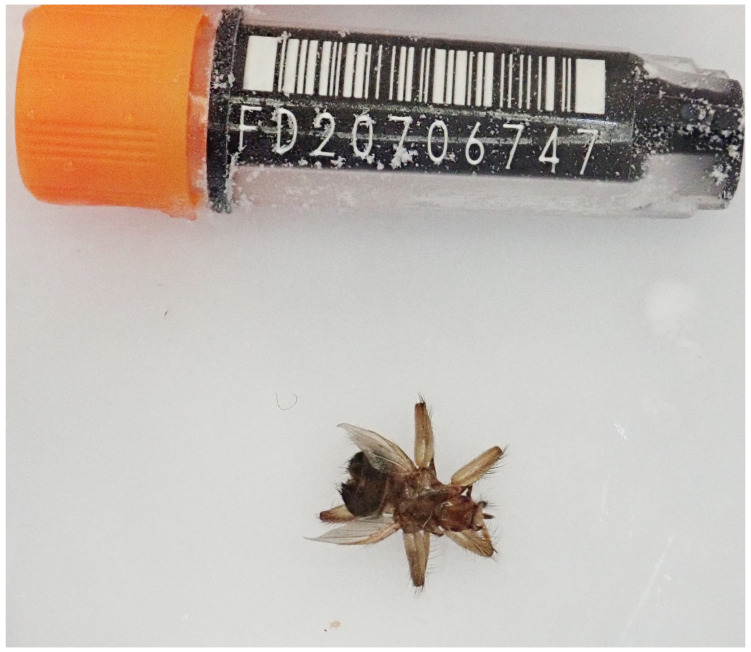
Photograph of the *Crataerina pallida* (idCraPall2) specimen used for genome sequencing.

The genome was sequenced from one *Crataerina pallida* ( Figure 1) collected from the Oxford University Museum of Natural History tower, Oxfordshire, UK (51.76, –1.25). A total of 144-fold coverage in Pacific Biosciences single-molecule HiFi long reads was generated. Primary assembly contigs were scaffolded with chromosome conformation Hi-C data. Manual assembly curation corrected 51 missing joins or misjoins and removed 8 haplotypic duplications, reducing the assembly length by 0.6% and the scaffold number by 83.72%, and increasing the scaffold N50 by 69.36%.

**Figure 2.**
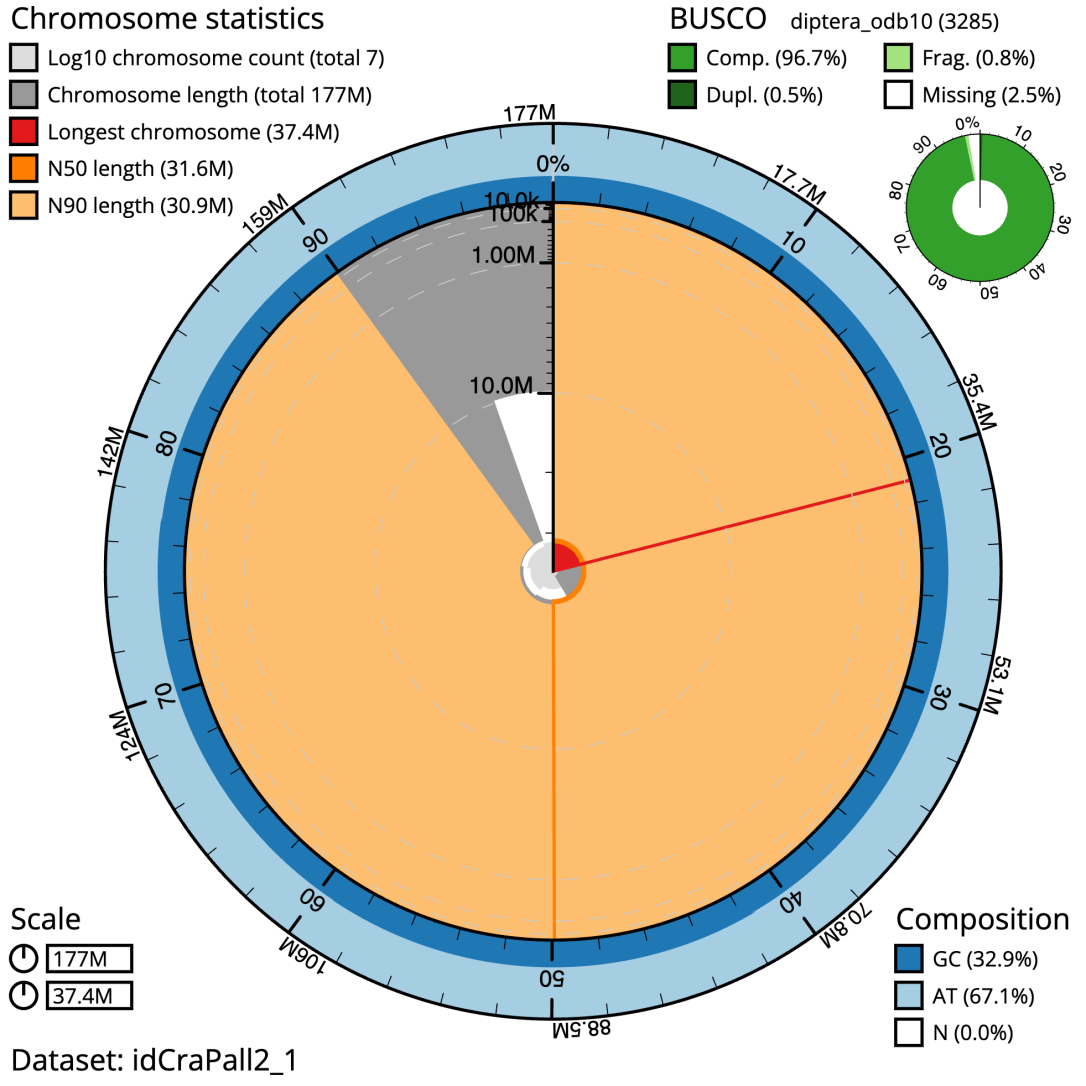
Genome assembly of *Crataerina pallida*, idCraPall2.1: metrics. The BlobToolKit Snailplot shows N50 metrics and BUSCO gene completeness. The main plot is divided into 1,000 size-ordered bins around the circumference with each bin representing 0.1% of the 177,070,084 bp assembly. The distribution of scaffold lengths is shown in dark grey with the plot radius scaled to the longest scaffold present in the assembly (37,350,342 bp, shown in red). Orange and pale-orange arcs show the N50 and N90 scaffold lengths (31,608,065 and 30,943,858 bp), respectively. The pale grey spiral shows the cumulative scaffold count on a log scale with white scale lines showing successive orders of magnitude. The blue and pale-blue area around the outside of the plot shows the distribution of GC, AT and N percentages in the same bins as the inner plot. A summary of complete, fragmented, duplicated and missing BUSCO genes in the diptera_odb10 set is shown in the top right. An interactive version of this figure is available at https://blobtoolkit.genomehubs.org/view/idCraPall2.1/dataset/idCraPall2_1/snail.

**Figure 3.**
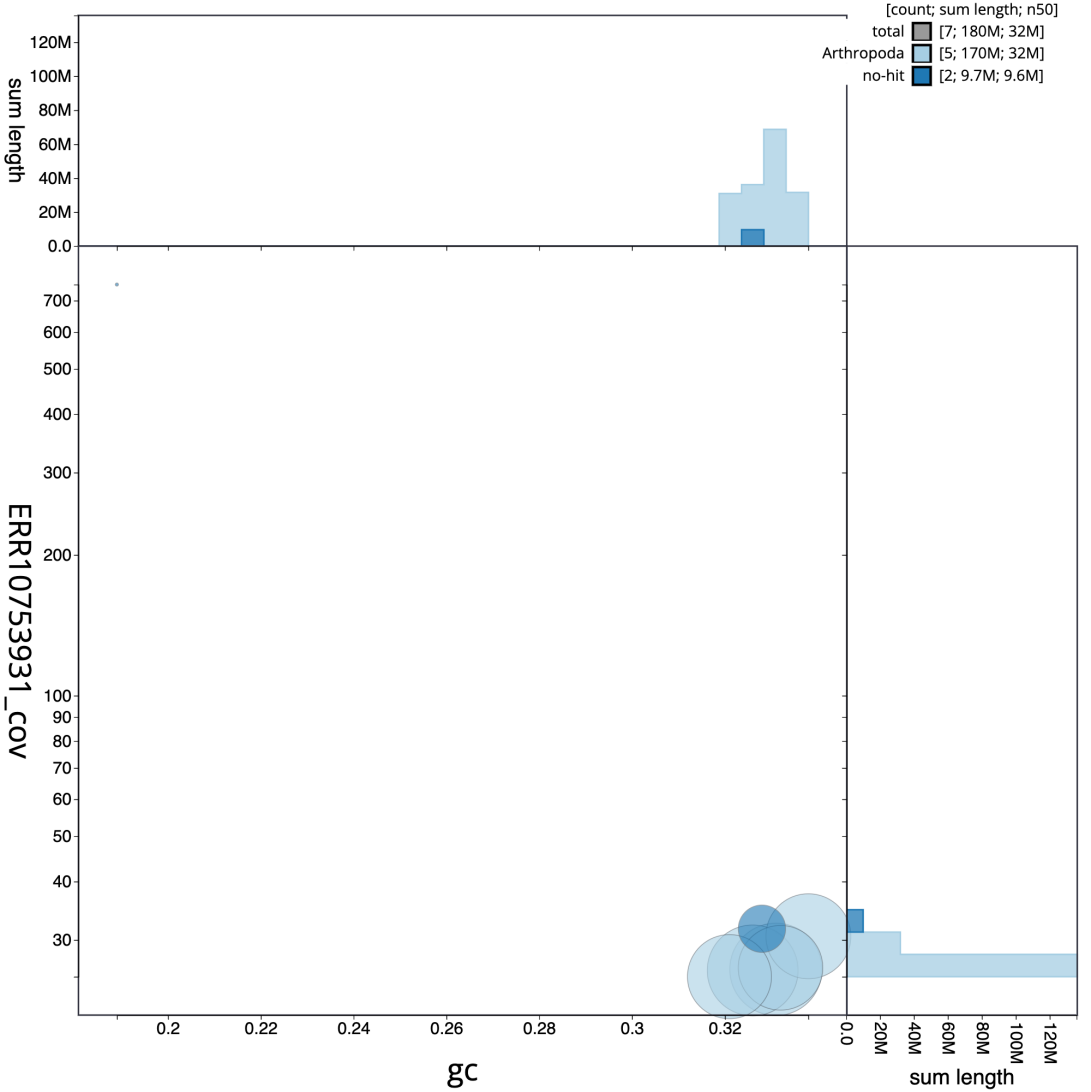
Genome assembly of *Crataerina pallida*, idCraPall2.1: BlobToolKit GC-coverage plot. Scaffolds are coloured by phylum. Circles are sized in proportion to scaffold length. Histograms show the distribution of scaffold length sum along each axis. An interactive version of this figure is available at https://blobtoolkit.genomehubs.org/view/idCraPall2.1/dataset/idCraPall2_1/blob.

**Figure 4.**
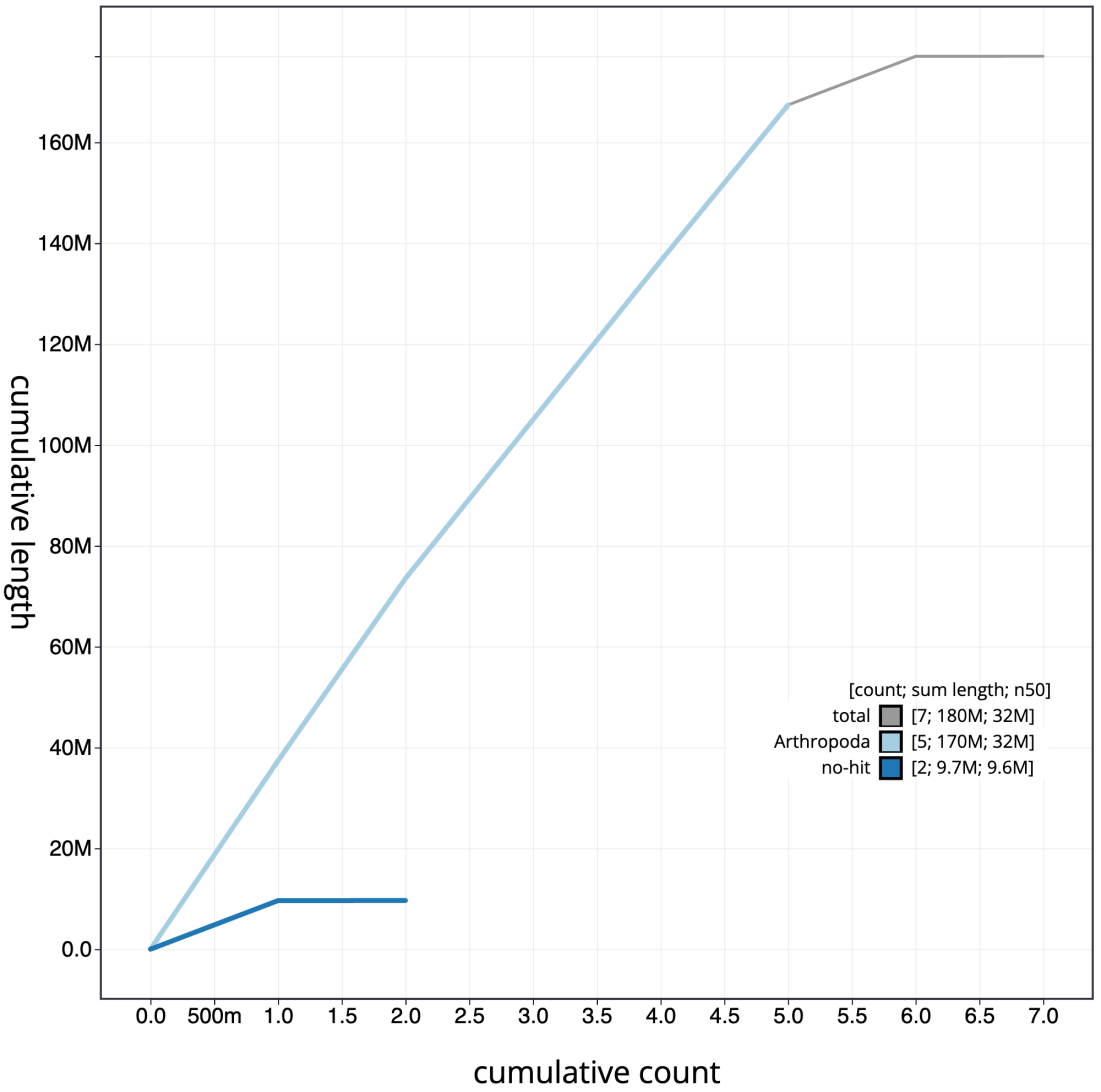
Genome assembly of *Crataerina pallida*, idCraPall2.1: BlobToolKit cumulative sequence plot. The grey line shows cumulative length for all scaffolds. Coloured lines show cumulative lengths of scaffolds assigned to each phylum using the buscogenes taxrule. An interactive version of this figure is available at https://blobtoolkit.genomehubs.org/view/idCraPall2.1/dataset/idCraPall2_1/cumulative.

**Figure 5.**
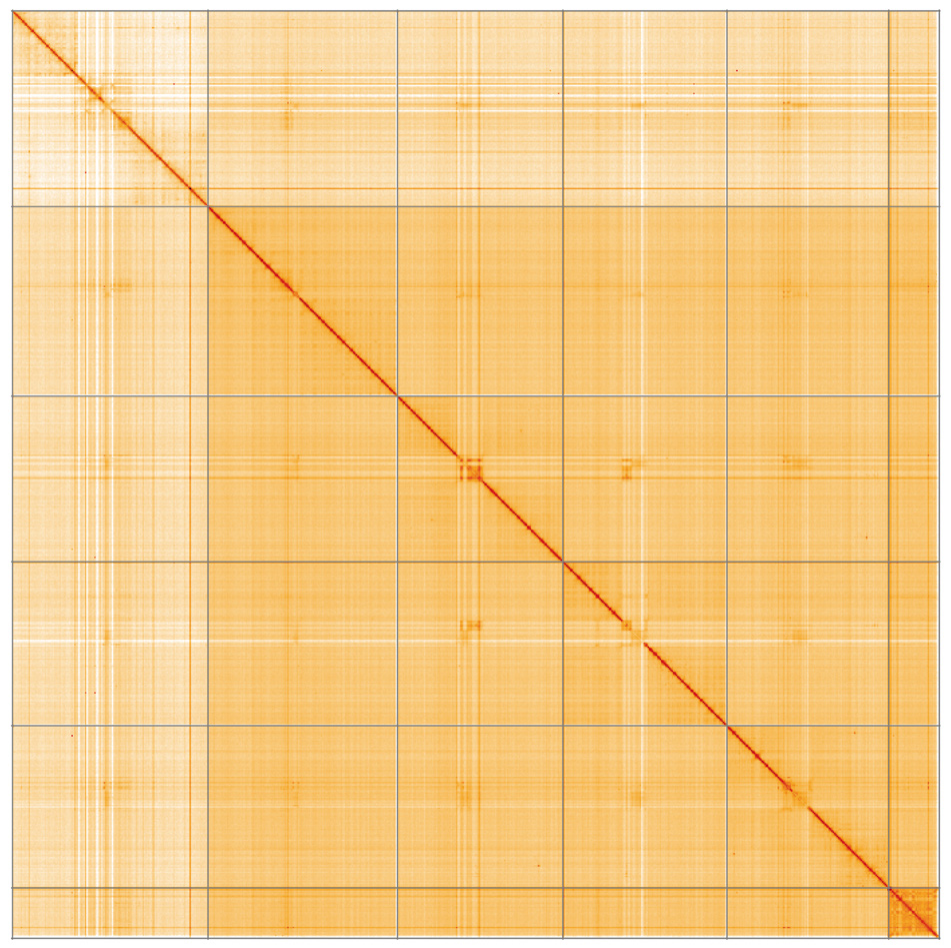
Genome assembly of *Crataerina pallida*, idCraPall2.1: Hi-C contact map of the idCraPall2.1 assembly, visualised using HiGlass. Chromosomes are shown in order of size from left to right and top to bottom. An interactive version of this figure may be viewed at https://genome-note-higlass.tol.sanger.ac.uk/l/?d=SK3mzTOGRDukmxsOjOrbIg.

The final assembly has a total length of 177.0 Mb in 6 sequence scaffolds with a scaffold N50 of 31.6 Mb ( Table 1). The snailplot in Figure 2 provides a summary of the assembly statistics, while the distribution of assembly scaffolds on GC proportion and coverage is shown in Figure 3. The cumulative assembly plot in Figure 4 shows curves for subsets of scaffolds assigned to different phyla. Most (99.98%) of the assembly sequence was assigned to 6 chromosomal-level scaffolds, representing 5 autosomes and the X sex chromosome. Chromosome-scale scaffolds confirmed by the Hi-C data are named in order of size ( Figure 5; Table 2). While not fully phased, the assembly deposited is of one haplotype. Contigs corresponding to the second haplotype have also been deposited. The mitochondrial genome was also assembled and can be found as a contig within the multifasta file of the genome submission.

**Table 1.** Genome data for *Crataerina pallida*, idCraPall2.1.

Project accession data		
Assembly identifier	idCraPall2.1	
Species	*Crataerina pallida*	
Specimen	idCraPall2	
NCBI taxonomy ID	452744	
BioProject	PRJEB58667	
BioSample ID	SAMEA10166867	
Isolate information	idCraPall2, female: head and thorax (DNA sequencing) idCraPall1, male: head and thorax (Hi-C scaffolding)	
Assembly metrics *		*Benchmark*
Consensus quality (QV)	67.9	*≥ 50*
*k*-mer completeness	100%	*≥ 95%*
BUSCO **	C:96.7%[S:96.2%,D:0.5%], F:0.8%,M:2.5%,n:3,285	*C ≥ 95%*
Percentage of assembly mapped to chromosomes	99.98%	*≥ 95%*
Sex chromosomes	X chromosome	*localised homologous pairs*
Organelles	Mitochondrial genome assembled	*complete single alleles*
Raw data accessions		
PacificBiosciences SEQUEL II	ERR10753931	
Hi-C Illumina	ERR10742414	
Genome assembly		
Assembly accession	GCA_949710015.1	
*Accession of alternate haplotype*	GCA_949710075.1	
Span (Mb)	177.0	
Number of contigs	105	
Contig N50 length (Mb)	3.5	
Number of scaffolds	6	
Scaffold N50 length (Mb)	31.6	
Longest scaffold (Mb)	37.4	

* Assembly metric benchmarks are adapted from column VGP-2020 of “Table 1: Proposed standards and metrics for defining genome assembly quality” from (Rhie *et al.*, 2021). ** BUSCO scores based on the diptera_odb10 BUSCO set using v5.3.2. C = complete [S = single copy, D = duplicated], F = fragmented, M = missing, n = number of orthologues in comparison. A full set of BUSCO scores is available at https://blobtoolkit.genomehubs.org/view/idCraPall2.1/dataset/idCraPall2_1/busco.

**Table 2.** Chromosomal pseudomolecules in the genome assembly of *Crataerina pallida*, idCraPall2.

INSDC accession	Chromosome	Length (Mb)	GC%
OX453290.1	1	36.17	32.5
OX453291.1	2	31.61	34.0
OX453292.1	3	31.34	33.0
OX453293.1	4	30.94	32.0
OX453294.1	5	9.64	33.0
OX453289.1	X	37.35	33.0
OX453295.1	MT	0.02	19.0

The estimated Quality Value (QV) of the final assembly is 67.9 with *k*-mer completeness of 100%, and the assembly has a BUSCO v5.3.2 completeness of 96.7% (single = 96.2%, duplicated = 0.5%), using the diptera_odb10 reference set ( *n* = 3,285).

Metadata for specimens, spectral estimates, sequencing runs, contaminants and pre-curation assembly statistics can be found at https://links.tol.sanger.ac.uk/species/452744.

## Methods

### Sample acquisition and nucleic acid extraction

*Crataerina pallida* specimens were collected from the Oxford University Museum of Natural History tower, Oxfordshire, UK (latitude 51.76, longitude –1.25) on 2021-04-17 and 2021-06-17. The specimens were taken as puparia from swift nests by George Candelin (independent researcher) by potting. The specimens were hatched the following spring and identified by Denise Wawman (University of Oxford). They were then preserved on dry ice. The specimen used for DNA sequencing was idCraPall2 (specimen ID Ox001397), while idCraPall1 (specimen ID Ox001202) was used for Hi-C data.

DNA was extracted at the Tree of Life laboratory, Wellcome Sanger Institute (WSI). The idCraPall2 sample was weighed and dissected on dry ice with tissue set aside for Hi-C sequencing. Head and thorax tissue was disrupted using a Nippi Powermasher fitted with a BioMasher pestle. High molecular weight (HMW) DNA was extracted using the Qiagen MagAttract HMW DNA extraction kit. HMW DNA was sheared into an average fragment size of 12–20 kb in a Megaruptor 3 system with speed setting 30. Sheared DNA was purified by solid-phase reversible immobilisation using AMPure PB beads with a 1.8X ratio of beads to sample to remove the shorter fragments and concentrate the DNA sample. The concentration of the sheared and purified DNA was assessed using a Nanodrop spectrophotometer and Qubit Fluorometer and Qubit dsDNA High Sensitivity Assay kit. Fragment size distribution was evaluated by running the sample on the FemtoPulse system.

### Sequencing

Pacific Biosciences HiFi circular consensus DNA sequencing libraries were constructed according to the manufacturers’ instructions. DNA sequencing was performed by the Scientific Operations core at the WSI on the Pacific Biosciences SEQUEL IIe (HiFi) instrument. Hi-C data were also generated from head and thorax tissue of idCraPall1 using the Arima2 kit and sequenced on the Illumina NovaSeq 6000 instrument.

### Genome assembly, curation and evaluation

Assembly was carried out with Hifiasm ( Cheng *et al.*, 2021) and haplotypic duplication was identified and removed with purge_dups ( Guan *et al.*, 2020). The assembly was then scaffolded with Hi-C data ( Rao *et al.*, 2014) using YaHS ( Zhou *et al.*, 2023). The assembly was checked for contamination and corrected using the gEVAL system ( Chow *et al.*, 2016) as described previously ( Howe *et al.*, 2021). Manual curation was performed using gEVAL, HiGlass ( Kerpedjiev *et al.*, 2018) and Pretext ( Harry, 2022). The mitochondrial genome was assembled using MitoHiFi ( Uliano-Silva *et al.*, 2023), which runs MitoFinder ( Allio *et al.*, 2020) or MITOS ( Bernt *et al.*, 2013) and uses these annotations to select the final mitochondrial contig and to ensure the general quality of the sequence.

A Hi-C map for the final assembly was produced using bwa-mem2 ( Vasimuddin *et al.*, 2019) in the Cooler file format ( Abdennur & Mirny, 2020). To assess the assembly metrics, the *k*-mer completeness and QV consensus quality values were calculated in Merqury ( Rhie *et al.*, 2020). This work was done using Nextflow ( Di Tommaso *et al.*, 2017) DSL2 pipelines “sanger-tol/readmapping” ( Surana *et al.*, 2023a) and “sanger-tol/genomenote” ( Surana *et al.*, 2023b). The genome was analysed within the BlobToolKit environment ( Challis *et al.*, 2020) and BUSCO scores ( Manni *et al.*, 2021; Simão *et al.*, 2015) were calculated.

Table 3 contains a list of relevant software tool versions and sources.

**Table 3.** Software tools: versions and sources.

Software tool	Version	Source
BlobToolKit	4.1.7	https://github.com/blobtoolkit/blobtoolkit
BUSCO	5.3.2	https://gitlab.com/ezlab/busco
gEVAL	N/A	https://geval.org.uk/
Hifiasm	0.16.1-r375	https://github.com/chhylp123/hifiasm
HiGlass	1.11.6	https://github.com/higlass/higlass
Merqury	MerquryFK	https://github.com/thegenemyers/MERQURY.FK
MitoHiFi	2	https://github.com/marcelauliano/MitoHiFi
PretextView	0.2	https://github.com/wtsi-hpag/PretextView
purge_dups	1.2.3	https://github.com/dfguan/purge_dups
sanger-tol/genomenote	v1.0	https://github.com/sanger-tol/genomenote
sanger-tol/readmapping	1.1.0	https://github.com/sanger-tol/readmapping/tree/1.1.0
YaHS	1.2a	https://github.com/c-zhou/yahs

### Wellcome Sanger Institute – Legal and Governance

The materials that have contributed to this genome note have been supplied by a Darwin Tree of Life Partner. The submission of materials by a Darwin Tree of Life Partner is subject to the **‘Darwin Tree of Life Project Sampling Code of Practice’**, which can be found in full on the Darwin Tree of Life website here. By agreeing with and signing up to the Sampling Code of Practice, the Darwin Tree of Life Partner agrees they will meet the legal and ethical requirements and standards set out within this document in respect of all samples acquired for, and supplied to, the Darwin Tree of Life Project.

Further, the Wellcome Sanger Institute employs a process whereby due diligence is carried out proportionate to the nature of the materials themselves, and the circumstances under which they have been/are to be collected and provided for use. The purpose of this is to address and mitigate any potential legal and/or ethical implications of receipt and use of the materials as part of the research project, and to ensure that in doing so we align with best practice wherever possible. The overarching areas of consideration are:

•   Ethical review of provenance and sourcing of the material

•   Legality of collection, transfer and use (national and international)

Each transfer of samples is further undertaken according to a Research Collaboration Agreement or Material Transfer Agreement entered into by the Darwin Tree of Life Partner, Genome Research Limited (operating as the Wellcome Sanger Institute), and in some circumstances other Darwin Tree of Life collaborators.

## Data Availability

European Nucleotide Archive: *Crataerina pallida* (common swift louse fly). Accession number PRJEB58667; https://identifiers.org/ena.embl/PRJEB58667. ( Wellcome Sanger Institute, 2023) The genome sequence is released openly for reuse. The *Crataerina pallida* genome sequencing initiative is part of the Darwin Tree of Life (DToL) project. All raw sequence data and the assembly have been deposited in INSDC databases.: The genome will be annotated using available RNA-Seq data and presented through the Ensembl pipeline at the European Bioinformatics Institute. Raw data and assembly accession identifiers are reported in Table 1.

## Author information

Members of the University of Oxford and Wytham Woods Genome Acquisition Lab are listed here: https://doi.org/10.5281/zenodo.4789928.

Members of the Darwin Tree of Life Barcoding collective are listed here: https://doi.org/10.5281/zenodo.4893703.

Members of the Wellcome Sanger Institute Tree of Life programme are listed here: https://doi.org/10.5281/zenodo.4783585.

Members of Wellcome Sanger Institute Scientific Operations: DNA Pipelines collective are listed here: https://doi.org/10.5281/zenodo.4790455.

Members of the Tree of Life Core Informatics collective are listed here: https://doi.org/10.5281/zenodo.5013541.

Members of the Darwin Tree of Life Consortium are listed here: https://doi.org/10.5281/zenodo.4783558.
