# Supplementary material for: The genome sequence of the Swift Louse Fly Crataerina pallida (Latreille, 1812)
Source: Wellcome Open Res. 2023 Oct 9;8:434. [Version 1] doi: 10.12688/wellcomeopenres.20097.1 (PMC10618637; doi:10.12688/wellcomeopenres.20097.1)
Supplement: Peer review history

# Reviewer report: Reviewer response for version 1

**DOI:** 10.21956/wellcomeopenres.22256.r68416

**Published:** 2023-10-31

**Authors:** Héloïse Bastide

**Affiliations:** 1 Laboratoire Évolution, Génomes, Comportement et Écologie, CNRS, IRD, Université Paris-Saclay - Institut Diversité, Gif-sur-Yvette, France

In this paper, Wawman *et al.* have sequenced the genome of one *Crataerina pallida* female. They obtained a high-quality assembly of 6 chromosomal-level scaffolds, including the X chromosome. While the metrics presented here show a nearly complete genome assembly, to which I do not have much to comment, the interest one could have in this work is certainly broader than the sole generation of new sequence data. As the authors emphasize, no genome was available for the family Hippoboscidae until now and it could thus enlighten us on its evolutionary history through the phylogenetic position of *Crataerina pallida* among the Calyptratae.

 Moreover, some of the assembly statistics, like total length, could have been further investigated as compared to genome size of the most closely related available species. Is 177.0 Mb typical of the genome size of Calyptratae species? *Crataerina pallida* being indeed an haematophagous ectoparasite of birds, it would also be interesting to know how its genome size compares to other sequenced parasitic species that generally show a reduced genome size. On another hand, though I am aware that the authors plan to annotate this genome in another study, this would have had all its place in this paper to check if this ectoparasitic lifestyle has evolved through a reduction of the number of genes as well as a change in the amount and nature of repetitive sequences (transposons family for example).

Are sufficient details of methods and materials provided to allow replication by others?

Yes

Is the rationale for creating the dataset(s) clearly described?

Yes

Are the datasets clearly presented in a useable and accessible format?

Yes

Are the protocols appropriate and is the work technically sound?

Yes

Reviewer Expertise:

Population genetics, evolutionary genomics, insects.

I confirm that I have read this submission and believe that I have an appropriate level of expertise to confirm that it is of an acceptable scientific standard.

# Reviewer report: Reviewer response for version 1

**DOI:** 10.21956/wellcomeopenres.22256.r68413

**Published:** 2023-10-13

**Authors:** Jaakko L.O. Pohjoismäki

**Affiliations:** 1 Department of Environmental and Biological Sciences, University of Eastern Finland, Joensuu, Finland

The report by Denise Wawman and coworkers presents the genome of Crataerina pallida, based on a female fly. The species, although abundant in swift colonies, is not trivial to obtain and a nice addition to the growing number of reference genomes from the DToL. As pointed out in the introduction, it is also the first complete genome of a louse fly. It is unfortunate that the specimen was a female, as males represent the heterogametic sex in Diptera. Although the sexual dimorphism is not clear in this species, males could have been possible to differentiate from the females with relative ease, based on the terminal segments of the abdomen, especially when reared material was available.

 Of the European species of louse flies, *Crataerina pallida* could be mistaken with *C. hirundini*s. Although the photo of the specimen is not too detailed, it shows enough to confirm the species identity, which is further backed by the host information. I did, however, a pairwise BLAST of the mitochondrial genome against the species Cox1 barcode sequence, which differed by one nucleotide. It would be very informative, if such check was performed always by the genome team.

 The genome metrics present the typical high quality of the DToL pipeline, to which I have not much to comment. I do not see the gene annotations in the genome view, not even for the mitochondrial genome, but I assume these will become available (just noted at the end that data availability states that it will be annotated using RNA-seq). I understand the rush to publish, but these would be really interesting to see when browsing through the available genome.

 Overall, the published genome will be a nice resource for anybody interested in taxonomy and evolution of Diptera. The published sequences can also contain the genomes of potential pathogens that the louse fly had carried, providing interesting snapshot of this type of dark diversity.

Are sufficient details of methods and materials provided to allow replication by others?

Yes

Is the rationale for creating the dataset(s) clearly described?

Yes

Are the datasets clearly presented in a useable and accessible format?

Yes

Are the protocols appropriate and is the work technically sound?

Yes

Reviewer Expertise:

Molecular biology, genetics, taxonomy

I confirm that I have read this submission and believe that I have an appropriate level of expertise to confirm that it is of an acceptable scientific standard.

# Response

**Published:** 2023-10-13

**Authors:** Tree of Life Team Sanger

**Affiliations:** Wellcome Trust Sanger Institute, UK

Thank you for your review. In relation to your comment on DNA barcoding, we do in fact check the barcodes of all genomes within the Darwin Tree of Life project. This information is accessible via the link provided in the data note, in this case: https://links.tol.sanger.ac.uk/species/452744.
